# Prevalence and Antimicrobial Resistance of *Escherichia coli* Isolated from Chicken Carcasses in Romania: Zoonotic Potential and Public Health Impact

**DOI:** 10.3390/vetsci13030256

**Published:** 2026-03-09

**Authors:** Ionica Iancu, Sebastian Alexandru Popa, Alexandru Gligor, Vlad Iorgoni, Paula Nistor, Ionela Popa, Janos Degi, Kálmán Imre, Livia Stângă, Viorel Herman

**Affiliations:** 1 Department of Infectious Diseases and Preventive Medicine, Faculty of Veterinary Medicine, University of Life Sciences “King Mihai I” from Timişoara, 300645 Timişoara, Romania; ionica.iancu@usvt.ro (I.I.); vlad.iorgoni@usvt.ro (V.I.); paula.nistor@usvt.ro (P.N.); ionela.popa@usvt.ro (I.P.); janosdegi@usvt.ro (J.D.); viorelherman@usvt.ro (V.H.); 2 Department of Animal Production and Veterinary Public Health, Faculty of Veterinary Medicine, University of Life Sciences “King Mihai I” from Timişoara, 300645 Timişoara, Romania; alexandru.gligor@usvt.ro (A.G.); kalmanimre@usvt.ro (K.I.); 3 Discipline of Microbiology, Faculty of Medicine, “Victor Babes” University of Medicine and Pharmacy, Eftimie Murgu Square 2, 300041 Timişoara, Romania; stanga.livia@umft.ro; 4 Academy of Romanian Scientists (AOSR), Splaiul Independentei 54, 050094 Bucharest, Romania

**Keywords:** poultry, chicken carcasses, food safety, AMR, retail, Romania

## Abstract

Antimicrobial-resistant bacteria associated with poultry production pose an ongoing challenge for food safety and veterinary public health. The present study evaluated the prevalence, antimicrobial resistance phenotypes, and selected resistance genes of *E. coli* recovered from broiler chicken carcasses and cecal content in Romania. Over a 12-month period in 2024, a total of 444 samples were collected, including 300 carcasses obtained from slaughterhouses and retail outlets and 144 cecal samples collected at slaughterhouses. Isolates were recovered using standard microbiological procedures and confirmed through biochemical and automated identification systems. Antimicrobial susceptibility was assessed using a minimum inhibitory concentration–based automated platform, and extended-spectrum β-lactamase (ESBL) production was evaluated phenotypically. Target resistance genes were investigated by PCR. *E. coli* was identified in 36.0% of carcass samples and 64.6% of cecal samples. High resistance rates were observed for tetracycline (82.6%), ampicillin (68.3%), and trimethoprim–sulfamethoxazole (61.2%), while multidrug resistance occurred in 34.3% of isolates. ESBL production was detected in 11.1% of carcass isolates and 11.8% of cecal isolates and was associated with the presence of *bla*CTX-M. Additional resistance determinants, including *bla*TEM, *tet*A, *tet*B, *sul*1, *dfr*A1, and *aad*A1, were widely distributed among isolates from both sources. The results suggest that poultry carcasses may contribute to the dissemination of resistant and ESBL-producing *E. coli*, reflecting intestinal carriage and contamination during processing. Strengthened antimicrobial stewardship, systematic resistance monitoring, and improved hygiene practices throughout the poultry production chain are essential to reduce the public health impact of resistant bacteria.

## Simple Summary

Poultry represents a substantial share of global food consumption, which places its microbiological safety among the key concerns in public health. Throughout slaughtering and subsequent processing stages, chicken carcasses may be exposed to bacterial contamination, particularly from microorganisms that inhabit the intestinal tract of animals, including *Escherichia coli* (*E. coli*). Some strains of this bacterium have developed resistance to antibiotics, meaning that infections caused by them may be more difficult to treat. In this study, chicken carcasses marketed in Romania together with cecal samples collected from slaughterhouses were examined to determine how frequently *E. coli* was present and whether the detected bacteria were resistant to commonly used antibiotics. The bacterium was found in 36.0% of the analyzed carcasses and 64.6% of cecal samples, and many isolates showed resistance to multiple antibiotics. These findings underscore a possible risk to consumers, as contaminated poultry carcasses may facilitate exposure to resistant microorganisms when adequate hygiene and biosecurity measures are not consistently ensured. The results underline the importance of responsible antibiotic use in animal production, effective hygiene measures during poultry processing, and proper food handling practices to reduce the risk of spreading antibiotic-resistant bacteria.

## 1. Introduction

The contemporary global landscape is characterized by increasing interconnectedness and sustained population growth, both of which intensify the demand for safe, efficient, and sustainable food production systems. These pressures highlight the necessity of an integrated and multidisciplinary approach to address the principal challenges affecting modern livestock production [1,2]. Among these challenges, infectious pathogens and the accelerating emergence of antimicrobial resistance (AMR) represent critical threats, affecting animal health while also undermining food security and population-level health outcomes [3,4,5,6]. AMR, in particular, has been identified as one of the major global concerns of the 21st century, underscoring the need for coordinated strategies that encompass surveillance, prevention, and responsible resource management within the livestock sector [7,8,9].

In response to expanding consumption trends, the poultry sector has undergone substantial intensification in recent decades. However, accelerated production dynamics may compromise the consistent implementation of biosecurity measures across the supply chain [10,11]. As a consequence, poultry carcasses can become contaminated with various pathogenic microorganisms, including *E. coli* [12,13,14], *Salmonella* spp. [15,16,17] and *Campylobacter* spp. [18,19], during slaughtering and processing stages. The presence of *E. coli* on carcasses is particularly important due to the ability of some strains to infect humans and to carry antimicrobial resistance determinants. [20].

Altogether, these vulnerabilities within modern poultry production systems have increasingly drawn attention to antimicrobial resistance, a phenomenon that compromises treatment outcomes in veterinary and human medicine [21,22]. This phenomenon has become a critical global concern, as resistant pathogens compromise the effectiveness of standard therapeutic options, prolong the duration and severity of infections, and generate substantial economic and public health burdens. The development and spread of AMR are driven by a complex interplay of factors, including the extensive and sometimes indiscriminate use of antimicrobials in human medicine, veterinary practice, and agricultural production [23]. In intensive livestock systems, antimicrobials are frequently applied for therapeutic purposes, thereby creating selective pressures that favor the emergence and persistence of resistant strains. Beyond direct transmission, resistance determinants may be dispersed through environmental media, promoting exchange between animal hosts, human populations, and surrounding ecosystems. Within this context, resistant *E. coli* associated with poultry products represent a key area of concern due to their role as reservoirs of resistance genes with significant zoonotic potential [24,25].

Considering these aspects, the study evaluated the occurrence and resistance characteristics of *E. coli* recovered from broiler carcass and intestinal samples collected in Romania. The objective was to provide updated information on contamination patterns along the poultry processing chain and to describe the distribution of resistance phenotypes among the isolates. Special emphasis was placed on antimicrobials classified as critically important for clinical practice in both veterinary and human medicine. By integrating microbiological findings with epidemiological considerations, this work examines the potential zoonotic implications of resistant strains detected on poultry carcasses. The results offer further insight into the contribution of poultry production to the maintenance and spread of antimicrobial-resistant bacteria and may assist future risk assessment initiatives and food safety strategies at national level.

## 2. Materials and Methods

### 2.1. Study Design

A 12-month study was conducted over 2024 to assess the prevalence and antimicrobial resistance profiles of *E. coli* isolated from broiler chicken samples intended for human consumption in Romania. A total of 300 carcasses and 144 cecum samples were collected from slaughterhouses and retail outlets, including supermarkets and local food markets. Sampling was performed at regular monthly intervals, a design chosen to ensure temporal representativeness across the entire year and to minimize potential seasonal bias associated with fluctuations in production practices, environmental conditions, and bacterial contamination levels.

At each sampling site, carcasses were randomly selected from available batches at comparable points along the distribution pathway to limit variability related to retail handling and storage conditions. Cecal content samples were collected exclusively from slaughterhouses and were aseptically obtained from slaughtered birds to investigate the intestinal carriage of *E. coli* and to allow comparison with isolates recovered from carcass surface samples. Collected material was preserved at 4 °C during transfer and processed microbiologically within a 24 h interval. Sampling focused on the external surfaces of the carcasses and consisted of the aseptic excision of approximately 10 g of neck skin from each carcass, in accordance with standard microbiological sampling procedures. These samples were collected to determine the presence and antimicrobial resistance patterns of *E. coli* associated with carcass surface contamination. All sampling procedures were performed under aseptic conditions to prevent cross-contamination. Collected material was transferred into an appropriate medium to allow standardized sample preparation for microbiological analysis, thereby ensuring consistency and comparability across sampling periods, production sources, and retail settings.

### 2.2. Bacterial Isolation and Identification from Broiler Carcasses

Neck skin samples were transferred into sterile 0.9% saline solution to obtain an initial ten-fold dilution. Approximately 10 g of neck skin was homogenized in sterile saline (0.9%) to obtain a primary 1:10 dilution. Serial dilutions were prepared, and aliquots were inoculated onto Violet Red Bile Glucose (VRBG; Oxoid Ltd., Basingstoke, UK) agar for selective recovery of Enterobacteriaceae. Plates were incubated at 37 °C for 24 h, and characteristic colonies were subsequently counted.

From each VRBG plate, 5–10 morphologically typical colonies were subcultured for identification and susceptibility testing. Purity was achieved by streaking onto non-selective agar and incubating at 37 °C for 24 h. Isolates were then plated on Tryptone Bile X-Glucuronide (TBX; Oxoid, UK) and Eosin Methylene Blue (Levine; Oxoid, UK) media and incubated under the respective conditions to assess characteristic growth features.

Following purification, only isolates exhibiting β-glucuronidase activity and indole positivity, attributes consistent with *E. coli*, were retained for further analysis. Presumptive *E. coli* colonies, identified by their blue-green appearance at the 10^−3^ dilution on TBX agar (Oxoid Ltd., Basingstoke, UK), were enumerated according to ISO 16649-2:2007 [26] and examined microscopically using Gram staining.

Presumptive *E. coli* isolates were initially characterized by Gram staining and routine biochemical assays. Species confirmation was obtained using the API 20E system and the VITEK^®^ 2 Compact platform (BioMérieux, Marcy l’Etoile, France) equipped with ID-GN cards. All procedures followed the manufacturer’s guidelines. *E. coli* ATCC 25922 served as the quality control strain.

To prevent overrepresentation of isolates originating from the same sample, one non-duplicate *E. coli* isolate per positive carcass was selected for subsequent antimicrobial susceptibility testing.

### 2.3. Cecal Content Sampling

In addition to carcass surface sampling, cecal content samples were collected to investigate the intestinal reservoir of *E. coli* and to better characterize potential sources of carcass contamination during slaughter and processing.

Specifically, cecum samples were aseptically collected from a subset of sampled birds immediately after evisceration, resulting in a total of 144 cecum samples.

Approximately 1 g of cecal content was aseptically collected and placed into sterile containers. Specimens were maintained at ~4 °C during transport and analyzed within 24 h. Each sample was diluted in 0.9% sterile saline solution to prepare a primary 1:10 suspension, followed by sequential dilutions. Portions of the diluted material were inoculated onto VRBG agar (Oxoid, Basingstoke, Hampshire, UK) and incubated at 37 °C for 24 h.

Presumptive *E. coli* isolates obtained from cecal samples were confirmed following the identification procedures previously described for carcass isolates (API 20E and VITEK^®^ 2 Compact). For subsequent susceptibility testing, a single representative isolate was retained from each positive sample.

### 2.4. Antimicrobial Susceptibility Test

Antimicrobial susceptibility testing of the confirmed *E. coli* isolates was performed using the Vitek 2 automated system (BioMérieux, Marcy-l’Étoile, France) with the AST-GN card, which contains a standardized panel of antimicrobial agents for Gram-negative bacteria. A fresh pure culture of each isolate was suspended in 0.9% saline solution to achieve a 0.5 McFarland turbidity standard, and the prepared inoculum was loaded into the instrument following the manufacturer’s instructions. The antimicrobial agents included in the routine susceptibility analysis were: β-lactams—ampicillin (AMP; 1, 4, 8, 32 µg/mL), amoxicillin/clavulanic acid (AMC; 4/2, 16/8, 32/16 µg/mL), and cefoperazone (CFP; 4, 8, 32 µg/mL); first-generation cephalosporins—cefalexin (LEX; 8, 32, 64 µg/mL) and cefalotin (CET; 2, 8, 32 µg/mL); third- and fourth-generation cephalosporins—ceftiofur (TIO; 1, 2 µg/mL) and cefquinome (CEF; 0.5, 1.5, 4 µg/mL); aminoglycosides—gentamicin (GEN; 4, 16, 32 µg/mL) and neomycin (NEO; 8, 16, 64 µg/mL); phenicols—florfenicol (FFC; 1, 4, 8 µg/mL); fluoroquinolones—enrofloxacin (ENR; 0.25, 1, 4 µg/mL) and marbofloxacin (MBX; 1, 2 µg/mL); quinolones—flumequine (UB; 2, 4, 8 µg/mL); sulfonamides—trimethoprim–sulfamethoxazole (SXT; 1/19, 4/76, 16/304 µg/mL); tetracyclines—tetracycline (TET; 2, 4, 8 µg/mL); and polymyxins—polymyxin B (PB; 0.25, 1, 4, 16 µg/mL). The system automatically monitored bacterial growth kinetics to determine minimum inhibitory concentrations (MICs), and results were interpreted according to CLSI (version 2024) and EUCAST (version 14.0; 2024) clinical breakpoints to classify isolates as susceptible, intermediate (susceptible, increased exposure for EUCAST), or resistant. *E. coli* ATCC 25922 was used as the quality-control strain to ensure the accuracy and validity of the susceptibility testing.

Extended-spectrum β-lactamase production was assessed using the automated ESBL screening module integrated into the VITEK^®^ 2 AST-GN card. Results were recorded qualitatively (presence or absence) and were not included in the routine antimicrobial susceptibility table.

### 2.5. Detection of AMR Genes

To determine the genetic determinants underlying the observed phenotypic resistance profiles, confirmed *E. coli* isolates recovered from carcass surface and paired cecal content samples were screened by polymerase chain reaction (PCR) for selected antimicrobial resistance genes.

Genomic DNA was obtained by heat lysis. Bacterial colonies were resuspended in sterile nuclease-free water, subjected to boiling for 10 min, and centrifuged at 12,000× *g* for 5 min. The resulting supernatant served as DNA template and was stored at −20 °C until further analysis.

PCR assays targeted genes commonly associated with resistance to β-lactams, tetracyclines, aminoglycosides, sulfonamides/trimethoprim, and extended-spectrum β-lactamases. The following genes were investigated: *bla*TEM, *tet*(A), *tet*(B), *aad*A1, *dfr*A1, *sul*1, and *bla*CTX-M.

PCRs were carried out in a total volume of 25 µL using 2× PCR Master Mix (Thermo Fischer Scientific, Vilnius, Lithuania), gene-specific primers (0.5 µM each), and 2 µL of DNA template. Thermal cycling included an initial denaturation step at 95 °C for 5 min, followed by 35 amplification cycles consisting of denaturation (94 °C, 30 s), primer annealing at gene-specific temperatures (30 s), and extension at 72 °C for 45 s, with a final extension at 72 °C for 5 min.

Amplified products were resolved by agarose gel electrophoresis (1.5%) and stained with GelRed (Biotium, Fremont, CA, USA). DNA fragments were visualized under ultraviolet light and sized using a 100 bp molecular weight marker. Appropriate positive and negative controls were included in all assays.

Detailed primer information and expected amplicon sizes are provided in Table 1.

### 2.6. Statistical Analysis

Statistical processing was conducted in GraphPad Prism 10.0.2 (GraphPad Software, Boston, MA, USA). Results are reported as proportions with 95% confidence intervals. Associations between sample origin and contamination rates were examined using Fisher’s exact test. A two-tailed *p*-value below 0.05 was considered statistically significant.

## 3. Results

### 3.1. Occurrence of E. coli in Carcass and Cecal Samples

The prevalence of *E. coli* varied according to sampling source and sample type as shown in Table 2. The highest proportion of positive samples (93/144; 64.6%) was observed in cecal content collected at the slaughterhouse level, whereas lower prevalence rates were recorded for carcass surface samples from both slaughterhouse (63/162; 38.9%) and retail settings (45/138; 32.6%).

### 3.2. Antimicrobial Susceptibility Testing

Antimicrobial susceptibility testing presented in Table 3, revealed high levels of resistance among *E. coli* isolates to several commonly used antimicrobial agents. The highest resistance rates were observed for tetracycline (82.6%), ampicillin (68.3%), and trimethoprim–sulfamethoxazole (61.2%).

Moderate resistance levels were detected against fluoroquinolones and quinolones, with resistance to enrofloxacin and neomycin exceeding 40%, while identified resistance to flumequine and marbofloxacin exceeded 30%. In contrast, third- and fourth-generation cephalosporins, including ceftiofur and cefquinome, retained high activity, with susceptibility rates above 85%. Polymyxin B showed the greatest effectiveness overall, with 92.5% of isolates classified as susceptible. Resistance to aminoglycosides was comparatively lower, particularly for gentamicin (12.0%), although elevated resistance to neomycin was observed.

Resistance to at least one antimicrobial agent across three or more distinct antimicrobial classes (multidrug resistance—MDR) was observed in 69 of the 201 isolates (34.3%). The results are presented in Table 4. Also, phenotypic screening for ESBL production detected 23 ESBL-positive isolates (11.4% overall), including 12 carcass and 11 cecal isolates.

### 3.3. Molecular Detection of AMR Genes

PCR screening identified multiple antimicrobial resistance determinants among *E. coli* isolates recovered from both carcass surface and cecal content samples. The distribution and prevalence are summarized in Table 5.

The β-lactam resistance gene *bla*TEM was detected in 74/108 (68.5%) carcass isolates and 67/93 (72.1%) cecal isolates. The ESBL-associated *bla*CTX-M gene was identified in 12/108 (11.1%) carcass isolates and 11/93 (11.8%) cecal isolates, corresponding to isolates previously classified as ESBL-positive by phenotypic testing.

Tetracycline resistance genes *tet*(A) and *tet*(B) were detected in 89/108 (82.6%) carcass isolates and 77/93 (82.8%) cecal isolates. Trimethoprim–sulfamethoxazole resistance determinants were also frequently observed. The *sul*1 gene was detected in 66/108 (61.1%) carcass isolates and 64/93 (68.9%) cecal isolates, while *dfr*A1 was detected in 61/108 (56.5%) and 54/93 (58.1%) isolates, respectively. The aminoglycoside resistance gene *aad*A1 was identified in 44/108 (40.7%) carcass isolates and 43/93 (46.2%) cecal isolates.

Most multidrug-resistant isolates carried two or more resistance genes simultaneously.

### 3.4. Comparative Analysis of E. coli Prevalence

The prevalence of *E. coli* on carcass surfaces was comparable between slaughterhouse samples (38.9%) and retail samples (32.6%), with no statistically significant difference detected between the two sources (*p* = 0.279).

At the slaughterhouse level, the prevalence of *E. coli* was significantly higher in cecal samples (64.6%) than on carcass surfaces (38.9%), indicating a substantially greater frequency of intestinal carriage compared with surface contamination at this stage of production (*p* < 0.001). When all sampling points were considered together, *E. coli* prevalence remained markedly higher in cecal samples than in carcass samples overall (64.6% vs. 36.0%), with this difference being statistically significant (*p* < 0.001).

## 4. Discussion

The present study provides updated data on the prevalence and antimicrobial resistance patterns of *E. coli* isolated from chicken carcasses marketed in Romania and contributes to a more refined understanding of antimicrobial resistance at the interface between poultry production and veterinary public health. The detection of *E. coli* in 36.0% of examined carcasses indicates that poultry carcasses remains a relevant reservoir of enteric bacteria and reflects the level of fecal contamination that may occur along the slaughtering and processing continuum. Such contamination can arise from multiple points in the production chain, including evisceration, carcass handling, and equipment hygiene, and may persist into the retail stage [33,34].

From a food safety perspective, the presence of *E. coli* on carcass surfaces is widely recognized as an indicator of poor hygienic performance and inadequate process control within poultry processing systems [35]. Although most isolates are commensal, their detection on carcasses intended for human consumption is of concern due to their capacity to act as carriers of antimicrobial resistance determinants [36]. Consequently, inadequate hygienic practices during slaughter, processing, distribution, or domestic food handling may facilitate the transmission of resistant bacteria to consumers, underscoring the relevance of continuous monitoring and targeted interventions throughout the poultry production chain [37].

The antimicrobial resistance profiles observed in this study were characterized by notably high resistance rates to tetracycline (82.6%), ampicillin (68.5%), and trimethoprim–sulfamethoxazole (61.1%). This resistance pattern aligns with findings commonly reported for poultry-associated *E. coli* across different production systems and geographic regions [33,38,39,40], suggesting a persistent and widespread selective pressure within the poultry sector. The elevated resistance to these agents likely reflects their historical use, and in some contexts continued application, in poultry production for therapeutic and prophylactic purposes [41,42].

Tetracyclines and penicillin, in particular, have been extensively employed in veterinary medicine owing to their broad-spectrum activity, oral availability, and relatively low cost [43,44]. Prolonged exposure to these antimicrobial classes has been associated with the maintenance and dissemination of resistance determinants, often located on mobile genetic elements that facilitate horizontal gene transfer. Similarly, resistance to trimethoprim–sulfamethoxazole may be linked to the long-standing use of folate pathway inhibitors in food-producing animals, further contributing to the persistence of multidrug-resistant *E. coli* populations within the poultry production environment [45].

The resistance patterns observed in the present study are consistent with those reported in recent investigations conducted in other animal production systems. Similar high resistance rates to tetracyclines, penicillins, and folate pathway inhibitors have been documented in livestock-associated *E. coli* worldwide. For example, Custódio et al. reported resistance rates of 74% to tetracycline and 62% to ampicillin among calf isolates in Brazil, with a high proportion of multidrug-resistant strains. In comparison, our poultry isolates showed comparable resistance trends, with 82.6% resistance to tetracycline and 68.3% to ampicillin, confirming that these antimicrobial classes remain under strong selective pressure across different animal species and production environments [46].

Importantly, the molecular analyses performed in the present study supported the phenotypic findings and provided further insight into the genetic basis of resistance. The high prevalence of *tet*(A) and *tet*(B) genes corresponded closely with the elevated tetracycline resistance rates, while frequent detection of *bla*TEM explained the widespread ampicillin resistance. Likewise, the presence of *sul1* and *dfrA1* genes paralleled the resistance observed to trimethoprim–sulfamethoxazole. Detection of *bla*CTX-M among ESBL-positive isolates confirmed the circulation of extended-spectrum β-lactamase determinants. The similar distribution of these genes in both cecal and carcass isolates further suggests that carcass contamination largely reflects intestinal carriage rather than exclusively environmental sources during processing.

Although the proportion of multidrug-resistant (MDR) isolates in the present study (34.3%) was lower than that reported in some bovine or poultry production systems, the occurrence remains epidemiologically significant and indicates the persistent circulation of resistant strains within the poultry production chain [47]. The presence of MDR *E. coli* in poultry carcasses represents a substantial concern for veterinary public health, as such strains may compromise therapeutic efficacy in both animal and human infections and serve as reservoirs of transferable resistance determinants [47,48].

The MDR prevalence observed in this study is consistent with reports from several regions worldwide, where MDR rates in *E. coli* isolated from chicken meat frequently exceed 60% and, in some cases, approach or surpass 90% [49,50,51,52]. These elevated figures have been attributed to differences in antimicrobial usage practices, regulatory frameworks, and production intensification. In contrast, earlier studies conducted in Romania reported considerably lower MDR proportions, approximately 17%, although these investigations were based on smaller sample sizes and relied on disk diffusion methods for susceptibility testing. Such methodological differences, including sampling design, analytical approach, and antimicrobial panels used, likely contribute to variability in reported MDR rates and underscore the importance of standardized surveillance protocols when comparing resistance data across studies and regions.

Despite variation in study design and analytical methods, resistance to ampicillin, tetracycline, and sulfonamides consistently emerges as the dominant MDR profile among poultry-associated *E. coli*. This recurring pattern, documented across multiple geographic regions, reflects sustained selective pressure exerted by long-standing antimicrobial use in poultry production and, in some cases, the accumulation of resistance determinants within single isolates.

The identification of ESBL-producing *E. coli* in 11.1% of isolates further highlights the relevance of poultry carcasses as a potential vehicle for resistance to critically important β-lactam antimicrobials. Although ESBL production was initially evaluated phenotypically, the molecular detection of *bla*CTX-M genes strengthens the evidence for the dissemination of extended-spectrum cephalosporin resistance through the food chain. Similar observations have been reported elsewhere, emphasizing the need for sustained surveillance of ESBL-producing *E. coli* within poultry production systems [53,54,55].

The resistance trends identified in the present investigation correspond to findings documented within European antimicrobial resistance monitoring systems managed by European Food Safety Authority (EFSA) and the European Centre for Disease Prevention and Control (ECDC). Recent EU summary reports consistently describe high levels of resistance in *E. coli* from broilers and poultry meat to tetracyclines, penicillins, and sulfonamides in many Member States, whereas resistance to aminoglycosides and polymyxins generally remains low. In addition, the recurring detection of multidrug-resistant and ESBL-producing *E. coli* in poultry has been identified as a continuing concern at the European level [56,57].

Within this framework, the findings from Romania fit within the broader European resistance landscape and further underline the contribution of poultry production to the dissemination of antimicrobial-resistant *E. coli*. Variability in resistance frequencies among countries is likely influenced by differences in antimicrobial usage policies, production intensity, biosecurity standards, and surveillance design. In this respect, the present results support EFSA recommendations calling for harmonized antimicrobial resistance monitoring in food-producing animals, as well as sustained efforts to reduce antimicrobial use in livestock through evidence-based stewardship initiatives.

In a broader European context, the prevalence observed in the present study (36.0% in carcasses and 64.6% in cecal samples) supports the well-established concept that the intestinal tract represents the primary reservoir of *E. coli*, while carcass contamination reflects transfer during processing. However, the carcass prevalence recorded in Romania appears higher than that reported in Northern European countries such as Denmark, where national surveillance data indicate lower contamination levels and more restricted resistance profiles, likely associated with long-standing prudent antimicrobial use policies [58,59]. By contrast, the prevalence and resistance patterns identified here resemble those described in Southern and Eastern European production systems, where *E. coli* colonization rates frequently exceed 50% and ESBL-producing strains are commonly reported across the broiler production pyramid [60]. Such regional variation is likely influenced by differences in antimicrobial usage, production intensity, and biosecurity implementation.

The multidrug resistance profiles observed in Romanian isolates are also consistent with global reports linking intensive poultry production with increased selection pressure for resistance to β-lactams and tetracyclines [61]. Although the specific distribution of resistance genes varies geographically, the persistence of MDR *E. coli* in broiler production remains a recognized public health concern worldwide.

Viewed through a One Health lens, the detection of multidrug-resistant and ESBL-producing *E. coli* on chicken carcasses illustrates the close interconnection between animal health, food safety, and human health. Veterinarians involved in poultry health management have a central role in promoting prudent antimicrobial use, optimizing flock health, and strengthening biosecurity measures. At the same time, improvements in hygiene during slaughter and processing, together with increased consumer awareness regarding safe food handling, remain essential components in limiting the foodborne transmission of antimicrobial-resistant bacteria.

Although the present study was designed to investigate the prevalence and antimicrobial resistance profiles of commensal and indicator *E. coli* isolates associated with chicken carcasses and cecal content, rather than to specifically target enterohemorrhagic *E. coli* (EHEC) serotypes, certain methodological considerations should be acknowledged. The isolation protocol, based on β-glucuronidase activity and incubation conditions consistent with standardized methods for indicator *E. coli* detection (ISO 16649-2), enables the reliable recovery of strains widely recognized as sentinel organisms for antimicrobial resistance surveillance and for evaluating potential zoonotic transmission pathways along the food chain. While this approach may not specifically target certain atypical pathogenic serotypes lacking β-glucuronidase activity, indicators of *E. coli* remain highly informative for assessing antimicrobial resistance dynamics and their public health relevance within poultry production systems. In addition, the selection of one non-duplicate isolate per positive sample, consistent with harmonized surveillance protocols, provides a robust and standardized estimate of antimicrobial resistance prevalence at the population level, while ensuring comparability with established monitoring frameworks although it may not fully capture the complete diversity of resistance phenotypes present within individual samples.

## 5. Conclusions

The findings of the present study demonstrate that chicken carcasses marketed in Romania constitute a relevant source of *E. coli* contamination, including strains exhibiting clinically significant antimicrobial resistance. The observed resistance patterns, characterized by high levels of resistance to commonly used antimicrobials and the occurrence of multidrug-resistant and ESBL-producing isolates, underscore the persistence of antimicrobial resistance within poultry production systems.

From a broader perspective, these results reinforce concerns regarding the role of poultry carcasses in the circulation of antimicrobial-resistant bacteria at the human–animal–environment interface. The consistency of the observed resistance profiles with European surveillance data highlights the importance of coordinated monitoring approaches and standardized methodologies for generating comparable resistance data across regions.

In line with a One Health framework, effective mitigation of antimicrobial resistance in the poultry sector requires integrated actions, including prudent antimicrobial use, improved flock health management, enhanced hygiene during slaughter and processing, and informed consumer practices. Continued surveillance and future studies incorporating molecular characterization of resistance determinants will be crucial for refining risk assessments and guiding evidence-based interventions.

## Figures and Tables

**Table 1:** Primers used for the detection of antimicrobial resistance genes.

Antimicrobial Class	Gene	Primer Sequence (5′–3′)	Amplicon Size (bp)	Annealing (°C)	Reference
β-lactams	*bla*TEM	F: TGGGTGCACGAGTGGGTTAC R: TTATCCGCCTCCATCCAGTC	526	58	[27]
Tetracyclines	*tet*(A)	F: GGTTCACTCGAACGACGTCA R: CTGTCCGACAAGTTGCATGA	577	50	[28]
Tetracyclines	*tet*(B)	F: CCTCAGCTTCTCAACGCGTG R: GCACCTTGCTGATGACTCTT	634	50	[28]
Aminoglycosides	*aad*A1	F: TATCCAGCTAAGCGCGAACT R: ATTTGCCGACTACCTTGGTC	447	58	[29]
Trimethoprim	*dfr*A1	F: GGAGTGCCAAAGGTGAACAGC R: GAGGCGAAGTCTTGGGTAAAAC	367	58	[30]
Sulfonamides	*sul*1	F: CGGCGTGGGCTACCTGAACG R: GCCGATCGCGTGAAGTTCCG	433	60	[31]
ESBL	*bla*CTX-M	F: ATGTGCAGYACCAGTAARGTKATGGC R: TGGGTRAARTARGTSACCAGAAYCAGCGG	593	60	[32]

**Table 2:** Prevalence of *Escherichia coli* in chicken samples from different sources.

Samples Origin	Samples Type	Number of Samples	Number of Positive (%)	95% CI
Slaughterhouse	Carcasses	162	63 (38.9)	31.7–46.6%
	Cecum	144	93 (64.6)	56.5–71.9%
Retail	Carcasses	138	45 (32.6)	25.4–40.8%
Total	n.a.	444	201 (45.3)	40.7–49.9%

Legend: n.a.—not applicable.

**Table 3:** Antimicrobial susceptibility profiles of *E. coli* isolates recovered from chicken carcasses and cecum (*n* = 201).

Antimicrobial Class	Antimicrobial Agent	Susceptible *n* (%)	Intermediate *n* (%)	Resistant *n* (%)
Penicillins	Ampicillin (AMP) ^a^	45 (22.4)	19 (9.3) *	137 (68.3)
β-lactam/β-lactamase inhibitor	Amoxicillin/clavulanic acid (AMC) ^a^	108 (53.7)	30 (14.9) *	63 (31.4)
1st-generation cephalosporin	Cefalexin (LEX) ^a^	115 (57.2)	34 (16.9) *	52 (25.9)
	Cefalotin (CET) ^b^	123 (61.3)	26 (12.9)	52 (25.8)
Cephalosporins	Cefoperazone (CFP) ^b^	134 (66.7)	30 (14.9)	37 (18.4)
4th-generation cephalosporin	Cefquinome (CEF) ^a^	175 (87.0)	11 (5.6) *	15 (7.4)
3rd-generation cephalosporin	Ceftiofur (TIO) ^a^	171 (85.2)	15 (7.4) *	15 (7.4)
Fluoroquinolones	Enrofloxacin (ENR) ^b^	82 (40.8)	37 (18.4)	82 (40.8)
	Marbofloxacin (MBX) ^b^	104 (51.8)	34 (16.9)	63 (31.3)
Quinolones	Flumequine (UB) ^b^	93 (46.3)	41 (20.4)	67 (33.3)
Aminoglycosides	Gentamicin (GEN) ^a^	160 (79.6)	17 (8.4) *	24 (12.0)
	Neomycin (NEO) ^b^	88 (43.8)	30 (14.9)	83 (41.3)
Tetracyclines	Tetracycline (TET) ^a^	26 (12.9)	9 (4.5) *	166 (82.6)
Phenicols	Florfenicol (FFC) ^b^	134 (66.7)	26 (12.9)	41 (20.4)
Sulfonamides	Trimethoprim/sulfamethoxazole (SXT) ^b^	63 (31.4)	15 (7.4)	123 (61.2)
Polymyxins	Polymyxin B (PB) ^b^	186 (92.5)	6 (3.0)	9 (4.5)

Legend: ^a^—interpreted according to EUCAST breakpoints; ^b^—interpreted according to CLSI breakpoints; *—Susceptible, increased exposure (EUCAST). According to EUCAST clinical breakpoint definitions, the “I” category indicates “Susceptible, increased exposure”, replacing the former “Intermediate” category.

**Table 4:** MDR profiles of *E. coli* isolates.

Resistance Classes	No. of Classes with Resistance	Antimicrobial Resistance Profile	No. of MDR Isolates (%)
β-lactams, Quinolones, Tetracyclines	3	AMP, ENR, TET	24 (34.8)
β-lactams, Quinolones, Aminoglycosides	3	AMP, ENR, NEO	18 (26.2)
Quinolones, Tetracyclines, Sulfonamides	3	MBX, UB, TET, SXT	11 (15.9)
β-lactams, Aminoglycosides, Tetracyclines	3	AMP, CET, NEO, TET	5 (7.2)
β-lactams, Quinolones, Aminoglycosides, Phenicols	4	LEX, MBX, NEO, FFC	2 (2.9)
β-lactams, Tetracyclines, Sulfonamides, Aminoglycosides	4	AMP, TET, SXT, NEO	4 (5.8)
Quinolones, Tetracyclines, Aminoglycosides, Sulfonamides, Phenicols	5	ENR, UB, TET, NEO, SXT, FFC	5 (7.2)

Legend: For MDR classification, penicillins and cephalosporins were grouped as β-lactams; quinolones and fluoroquinolones were grouped as quinolones. Multiple agents from the same antimicrobial class may be present within a single MDR profile.

**Table 5:** Prevalence of antimicrobial resistance genes among *E. coli* isolates from carcass surface (*n* = 108) and cecal content samples (*n* = 93).

Gene	Carcass (*n* = 108) *n* (%)	Cecum (*n* = 93) *n* (%)	Total *n* (%)
*bla*TEM	74 (68.5)	67 (72.1)	141 (70.1)
*bla*CTX-M	12 (11.1)	11 (11.8)	23 (11.4)
*tet*(A)/*tet*(B)	89 (82.4)	77 (82.8)	166 (82.6)
*sul*1	66 (61.1)	64 (68.9)	130 (64.7)
*dfr*A1	61 (56.5)	54 (58.1)	115 (57.2)
*aad*A1	44 (40.7)	43 (46.2)	87 (43.3)

## Data Availability

The original contributions presented in this study are included in the article. Further inquiries can be directed to the corresponding author.

## References

[1] Michalk D.L., Kemp D.R., Badgery W.B., Wu J., Zhang Y., Thomassin P.J. (2019). Sustainability and future food security—A global perspective for livestock production. Land Degrad. Dev..

[2] Rehman A., Ulucak R., Ma H., Ding J., Hua J. (2024). The Interconnectedness of Land–Crops–Livestock and Environmental Quality in Emerging Asian Economies: Challenges of Agriculturalization and Carbonization. Land.

[3] Brooks D.R., Hoberg E.P., Boeger W.A., Trivellone V. (2022). Emerging infectious disease: An underappreciated area of strategic concern for food security. Transbound. Emerg. Dis..

[4] Löscher T., Prüfer-Krämer L., Krämer A., Kretzschmar M., Krickeberg K. (2009). Emerging and Re-emerging Infectious Diseases. Modern Infectious Disease Epidemiology: Concepts, Methods, Mathematical Models, and Public Health.

[5] Ferri M., Ranucci E., Romagnoli P., Giaccone V. (2017). Antimicrobial resistance: A global emerging threat to public health systems. Crit. Rev. Food Sci. Nutr..

[6] George A. (2018). Antimicrobial resistance, trade, food safety and security. One Health.

[7] Murray C.J., Ikuta K.S., Sharara F., Swetschinski L., Aguilar G.R., Gray A., Han C., Bisignano C., Rao P., Wool E. (2022). Global burden of bacterial antimicrobial resistance in 2019: A systematic analysis. Lancet.

[8] Naddaf M. (2024). 40 million deaths by 2050: Toll of drug-resistant infections to rise by 70%. Nature.

[9] Woolhouse M., Ward M., van Bunnik B., Farrar J. (2015). Antimicrobial resistance in humans, livestock and the wider environment. Philos. Trans. R. Soc. Lond. B Biol. Sci..

[10] Solano-Blanco A.L., González J.E., Medaglia A.L. (2023). Production Planning Decisions in the Broiler Chicken Supply Chain with Growth Uncertainty. Oper. Res. Perspect..

[11] Sohail M.N., Isloor S., Rathnamma D., Chandra Priya S., Veeregowda B.M., Hegde N.R., Varga C., Williams N.J. (2025). Phenotypic and Genotypic Characterization of Colistin, ESBL, and Multidrug Resistance in *Escherichia coli*Across the Broiler Production Chain in Karnataka, India. Poultry.

[12] Lima-Filho J.V., Martins L.V., de Nascimento D.C.O., Ventura R.F., Batista J.E.C., Silva A.F.B., Ralph M.T., Vaz R.V., Rabello C.B.-V., de Matos Mendes da Silva I. (2013). Zoonotic potential of multidrug-resistant extraintestinal pathogenic *Escherichia coli* obtained from healthy poultry carcasses in Salvador, Brazil. Braz. J. Infect. Dis..

[13] Belluco S., Barco L., Roccato A., Ricci A. (2016). *Escherichia coli* and *Enterobacteriaceae* counts on poultry carcasses along the slaughterline: A systematic review and meta-analysis. Food Control.

[14] Rus A., Bucur I.M., Imre K., Tirziu A.T., Ivan A.A., Gros R.V., Moza A.C., Popa S.A., Ban-Cucerzan A., Tirziu E. (2025). Phenotypic and Genotypic Characterization of ESBL and AmpC β-Lactamase-Producing *E. coli*Isolates from Poultry in Northwestern Romania. Antibiotics.

[15] Donado-Godoy P., Clavijo V., Leon M., Tafur M.A., Gonzales S., Hume M., Alali W., Walls I., Lo Fo Wong D.M.A., Doyle M.P. (2012). Prevalence of Salmonella on retail broiler chicken meat carcasses in Colombia. J. Food Prot..

[16] Elshebrawy H.A., Abdel-Naeem H.H.S., Mahros M.A., Elsayed H., Imre K., Herman V., Morar A., Sallam K.I. (2022). Multidrug-resistant *Salmonella enterica* serovars isolated from frozen chicken carcasses. LWT.

[17] Gómez-Baltazar A., Godínez-Oviedo A., Segura-García L.E., Hernández-Pérez C.F., Hernández-Iturriaga M., Cabrera-Díaz E. (2024). Genomic Diversity of *Salmonella enterica* Isolated from Raw Chicken at Retail Establishments in Mexico. Int. J. Food Microbiol..

[18] Popa S.A., Morar A., Ban-Cucerzan A., Imre K. (2022). Last decade mini-review of the scientific progresses in the monitoring of the occurrence and antimicrobial susceptibility profile of poultry origin *Campylobacter* spp. within the European Union countries. Rev. Rom. Med. Vet..

[19] Popa S.A., Herman V., Tîrziu E., Morar A., Ban-Cucerzan A., Imre M., Pătrînjan R.-T., Imre K. (2025). Public Health Risk of *Campylobacter* spp. Isolated from Slaughterhouse and Retail Poultry Meat: Prevalence and Antimicrobial Resistance Profiles. Pathogens.

[20] Iancu I. (2019). Evaluation of Antimicrobial Resistance in Strains of *E. coli* Isolated from Broiler Carcasses. Rev. Rom. Med. Vet..

[21] Prestinaci F., Pezzotti P., Pantosti A. (2015). Antimicrobial resistance: A global multifaceted phenomenon. Pathog. Glob. Health.

[22] Tacconelli E., Sifakis F., Harbarth S., Schrijver R., van Mourik M., Voss A., Sharland M., Rajendran N.B., Rodríguez-Baño J., Bielicki J. (2018). Surveillance for control of antimicrobial resistance. Lancet Infect. Dis..

[23] Domínguez D.C., Meza-Rodriguez S.M. (2019). Development of antimicrobial resistance: Future challenges. Pharmaceuticals and Personal Care Products: Waste Management and Treatment Technology Emerging Contaminants and Micro Pollutants.

[24] Ewers C., Antão E.M., Diehl I., Philipp H.C., Wieler L.H. (2013). Intestine and environment of the chicken as reservoirs for extraintestinal pathogenic *Escherichia coli* strains with zoonotic potential. Appl. Environ. Microbiol..

[25] Joseph J., Zhang L., Adhikari P., Evans J.D., Ramachandran R. (2023). Avian Pathogenic *Escherichia coli* (APEC) in Broiler Breeders: An Overview. Pathogens.

[26] (2007). Microbiology of Food and Feeding Stuffs. Horizontal Method for the Detection and Enumeration of Escherichia coli β-glucuronidase Positive.

[27] Tenover F.C., Huang M.B., Rasheed J.K., Persing D.H. (1994). Development of Pcr Assays to Detect Ampicillin Resistance Genes in Cerebrospinal-Fluid Samples Containing Haemophilus-Influenzae. J. Clin. Microbiol..

[28] Randall L.P., Cooles S.W., Osborn M.K., Piddock L.J.V., Woodward M.J. (2004). Antibiotic Resistance Genes, Integrons and Multiple Antibiotic Resistance in Thirty-Five Serotypes of Salmonella Enterica Isolated from Humans and Animals in the UK. J. Antimicrob. Chemother..

[29] Van T.T., Chin J., Chapman T., Tran L.T., Coloe P.J. (2008). Safety of raw meat and shellfish in vietnam: An analysis of *Escherichia coli* isolations for antibiotic resistance and virulence genes. Int. J. Food Microbiol..

[30] Toro C.S., Farfán M., Contreras I., Flores O., Navarro N., Mora G.C., Prado V. (2005). Genetic Analysis of Antibiotic-Resistance Determinants in Multidrug-Resistant Shigella Strains Isolated from Chilean Children. Epidemiol. Infect..

[31] Aslam M., Checkley S., Avery B., Chalmers G., Bohaychuk V., Gensler G., Reid-Smith R., Boerlin P. (2012). Phenotypic and genetic characterization of antimicrobial resistance in *Salmonella* serovars isolated from retail meats in Alberta, Canada. Food Microbiol..

[32] Zong Z., Partridge S.R., Thomas L., Iredell J.R. (2008). Dominance of *bla*_CTX-M_ within an Australian Extended-Spectrum β-Lactamase Gene Pool. Antimicrob. Agents Chemother..

[33] Eltai N.O., Yassine H.M., El-Obeid T., Al-Hadidi S.H., Al Thani A.A., Alali W.Q. (2020). Prevalence of antibiotic-resistant *Escherichia coli* isolates from local and imported retail chicken carcasses. J. Food Prot..

[34] Klaharn K., Pichpol D., Meeyam T., Harintharanon T., Lohaanukul P., Punyapornwithaya V. (2022). Bacterial contamination of chicken meat in slaughterhouses and the associated risk factors: A nationwide study in Thailand. PLoS ONE.

[35] Brătfelan D.O., Tabaran A., Colobatiu L., Mihaiu R., Mihaiu M. (2023). Prevalence and Antimicrobial Resistance of *Escherichia coli* Isolates from Chicken Meat in Romania. Animals.

[36] Husna A., Rahman M.M., Badruzzaman A.T.M., Sikder M.H., Islam M.R., Rahman M.T., Alam J., Ashour H.M. (2023). Extended-Spectrum β-Lactamases (ESBL): Challenges and Opportunities. Biomedicines.

[37] Boysen L., Nauta M., Rosenquist H. (2016). Campylobacter spp. and Escherichia coli contamination of broiler carcasses across the slaughter line in Danish slaughterhouses. Microb. Risk Anal..

[38] Casella T., Nogueira M.C.L., Saras E., Haenni M., Madec J.-Y. (2017). High prevalence of ESBLs in retail chicken meat despite reduced use of antimicrobials in chicken production, France. Int. J. Food Microbiol..

[39] Hussain A., Shaik S., Ranjan A., Nandanwar N., Tiwari S.K., Majid M., Baddam R., Qureshi I.A., Semmler T., Wieler L.H. (2017). Risk of transmission of antimicrobial resistant Escherichia coli from commercial broiler and free-range retail chicken in India. Front. Microbiol..

[40] Sary K., Fairbrother J.M., Arsenault J., De Lagarde M., Boulianne M. (2019). Antimicrobial Resistance and Virulence Gene Profiles among *Escherichia coli* Isolates from Retail Chicken Carcasses in Vietnam. Foodborne Pathog. Dis..

[41] Ghimpețeanu O.M., Pogurschi E.N., Popa D.C., Dragomir N., Drăgotoiu T., Mihai O.D., Petcu C.D. (2022). Antibiotic Use in Livestock and Residues in Food—A Public Health Threat: A Review. Foods.

[42] De Mesquita Souza Saraiva M., Lim K., do Monte D.F.M., Givisiez P.E.N., Alves L.B.R., de Freitas Neto O.C., Kariuki S., Júnior A.B., de Oliveira C.J.B., Gebreyes W.A. (2022). Antimicrobial resistance in the globalized food chain: A One Health perspective applied to the poultry industry. Braz. J. Microbiol..

[43] Simoneit C., Burow E., Tenhagen B.-A., Käsbohrer A. (2015). Oral Administration of Antimicrobials Increase Antimicrobial Resistance in E. Coli from Chicken—A Systematic Review. Prev. Vet. Med..

[44] Popa S.A., Morar A., Ban-Cucerzan A., Tîrziu E., Herman V., Imre M., Florea T., Morar D., Pătrînjan R.-T., Imre K. (2024). First study in the frequency of isolation and phenotypic antimicrobial resistance profiles of pig and cattle origin *Campylobacter* strains in Romania. Vet. Res. Commun..

[45] Miles T.D., McLaughlin W., Brown P.D. (2006). Antimicrobial resistance of Escherichia coli isolates from broiler chickens and humans. BMC Vet. Res..

[46] Custódio D.A.d.C., Pereira C.R., Gonçalves M.S., Costa A.C.T.R.B., de Oliveira P.F.R., da Silva B.H.P., Carneiro G.B., Coura F.M., Lage A.P., Heinemann M.B. (2024). Antimicrobial Resistance and Public and Animal Health Risks Associated with Pathogenic *Escherichia coli* Isolated from Calves. Comp. Immunol. Microbiol. Infect. Dis..

[47] Shafiq M., Huang J., Shah J.M., Ali I., Rahman S.U., Wang L. (2021). Characterization and resistant determinants linked to mobile elements of ESBL-producing and mcr-1-positive *Escherichia coli* recovered from the chicken origin. Microb. Pathog..

[48] Rafiq K., Islam M.R., Siddiky N.A., Samad M.A., Chowdhury S., Hossain K.M.M., Rume F.I., Hossain M.K., Mahbub-E-Elahi A., Ali M.Z. (2022). Antimicrobial Resistance Profile of Common Foodborne Pathogens Recovered from Livestock and Poultry in Bangladesh. Antibiotics.

[49] Rahman M.M., Husna A., Elshabrawy H.A., Alam J., Runa N.Y., Badruzzaman A.T.M., Banu N.A., Al Mamun M., Paul B., Das S. (2020). Isolation and Molecular Characterization of Multidrug-Resistant Escherichia Coli from Chicken Meat. Sci. Rep..

[50] Parvin M., Talukder S., Ali M., Chowdhury E.H., Rahman M., Islam M. (2020). Antimicrobial Resistance Pattern of *Escherichia coli* Isolated from Frozen Chicken Meat in Bangladesh. Pathogens.

[51] Khanom H., Nath C., Mshelbwala P.P., Pasha M.R., Magalhaes R.S., Alawneh J.I., Hassan M.M. (2025). Epidemiology and Molecular Characterisation of Multidrug-Resistant *Escherichia Coli* Isolated from Chicken Meat. PLoS ONE.

[52] Ahmed Z.S., Hashad M.E., Atef Y., Badr H., Elhariri M., Kadry M. (2025). Public health threat of antimicrobial resistance and virulence genes in Escherichia coli from human-chicken transmission in Egypt. Sci. Rep..

[53] Stuart J.C., Munckhof T.V.D., Voets G., Scharringa J., Fluit A., Hall M.L.-V. (2012). Comparison of ESBL contamination in organic and conventional retail chicken meat. Int. J. Food Microbiol..

[54] Reich F., Schill F., Atanassova V., Klein G. (2016). Quantification of ESBL-*Escherichia coli* on broiler carcasses after slaughtering in Germany. Food Microbiol..

[55] Ramos S., Silva V., Dapkevicius M.d.L.E., Caniça M., Tejedor-Junco M.T., Igrejas G., Poeta P. (2020). *Escherichia coli*as Commensal and Pathogenic Bacteria among Food-Producing Animals: Health Implications of Extended Spectrum β-Lactamase (ESBL) Production. Animals.

[56] CDC (2019). Antibiotic Resistance Threats in the United States.

[57] European Food Safety Authority (EFSA), European Centre for Disease Prevention and Control (2025). The European Union Summary Report on Antimicrobial Resistance in Zoonotic and Indicator Bacteria from Humans, Animals and Food in 2022–2023. EFSA J..

[58] Poulsen L.L., Bisgaard M., Christensen H. (2023). Prevalence of Potential Pathogenic and Antimicrobial Resistant *Escherichia coli* in Danish Broilers. Antibiotics.

[59] DANMAP (2024). DANMAP 2024—Use of Antimicrobial Agents and Occurrence of Antimicrobial Resistance in Bacteria from Food Animals, Food and Humans in Denmark.

[60] Apostolakos I., Mughini-Gras L., Fasolato L., Piccirillo A. (2019). Assessing the occurrence and transfer dynamics of ESBL/pAmpC-producing *Escherichia coli* across the broiler production pyramid. PLoS ONE.

[61] Ferreira M., Leão C., Clemente L., Albuquerque T., Amaro A. (2022). Antibiotic Susceptibility Profiles and Resistance Mechanisms to β-Lactams and Polymyxins of *Escherichia coli* from Broilers Raised under Intensive and Extensive Production Systems. Microorganisms.

